# Diagnosis of Alzheimer’s disease utilizing amyloid and tau as fluid biomarkers

**DOI:** 10.1038/s12276-019-0250-2

**Published:** 2019-05-09

**Authors:** Jinny Claire Lee, Soo Jung Kim, Seungpyo Hong, YoungSoo Kim

**Affiliations:** 1 Integrated Science and Engineering Division, Yonsei University, Incheon, 21983 Republic of Korea; 2 Department of Pharmacy, Yonsei University, Incheon, 21983 Republic of Korea; 3 Yonsei Institute of Pharmaceutical Sciences, Yonsei University, Incheon, 21983 Republic of Korea; 4 Yonsei Frontier Lab, Yonsei University, Incheon, 21983 Republic of Korea; 5 Pharmaceutical Sciences Division, School of Pharmacy and Carbone Cancer Center, School of Medicine & Public Health, University of Wisconsin, Madison, WI 53705 USA

**Keywords:** Diagnostic markers, Alzheimer's disease

## Abstract

Current technological advancements in clinical and research settings have permitted a more intensive and comprehensive understanding of Alzheimer’s disease (AD). This development in knowledge regarding AD pathogenesis has been implemented to produce disease-modifying drugs. The potential for accessible and effective therapeutic methods has generated a need for detecting this neurodegenerative disorder during early stages of progression because such remedial effects are more profound when implemented during the initial, prolonged prodromal stages of pathogenesis. The aggregation of amyloid-β (Aβ) and tau isoforms are characteristic of AD; thus, they are considered core candidate biomarkers. However, research attempting to establish the reliability of Aβ and tau as biomarkers has culminated in an amalgamation of contradictory results and theories regarding the biomarker concentrations necessary for an accurate diagnosis. In this review, we consider the capabilities and limitations of fluid biomarkers collected from cerebrospinal fluid, blood, and oral, ocular, and olfactory secretions as diagnostic tools for AD, along with the impact of the integration of these biomarkers in clinical settings. Furthermore, the evolution of diagnostic criteria and novel research findings are discussed. This review is a summary and reflection of the ongoing concerted efforts to establish fluid biomarkers as a diagnostic tool and implement them in diagnostic procedures.

## Alzheimer’s disease: early diagnostic biomarkers in body fluids

Markers from body fluids could help clinicians diagnose Alzheimer’s disease before cognitive decline appears. After numerous setbacks in treating advanced Alzheimer’s, researchers are eager to identify biological indicators that facilitate earlier disease detection and interception. A review by YoungSoo Kim and colleagues at Yonsei University in South Korea, explores the promise of ‘fluid biomarkers,’ which enables diagnosis using cerebrospinal fluid (CSF), blood, oral, ocular, and olfactory fluid samples. Shifts in CSF levels of amyloid beta and tau, two proteins central to Alzheimer’s pathology, can reliably monitor at-risk individuals. Although CSF collection is unpleasant for patients, it remains more promising than blood, where current data for candidate fluid biomarkers are relatively inconclusive. In this review, investigations to discover safer, cheaper, and more reliable diagnostic tools to shift treatment from alleviation to prevention are introduced.

## Introduction

It is possible to observe neuritic plaques and neurofibrillary tangles within the brain tissue of patients afflicted by Alzheimer’s disease (AD). These neuropathological alterations often parallel the progression of cognitive impairment. Thus, AD pathology consists of amyloid-β (Aβ) deposition in the brain, the hyperphosphorylation of tau proteins, and neuroinflammation through glial activation^1^. Aβ peptides are often referred to by the length of their amino acid sequences and can be found in cerebral and peripheral tissues. Although there are many conformations of Aβ, which commonly consists of 36–43 amino acids, Aβ42 is known to aggregate the most readily and aid in the formation of neuritic plaques^2^. Another characteristic of AD is the presence of neuronal lesions composed of tau proteins. Studying these aggregated forms of hyperphosphorylated tau, also referred to as neurofibrillary tangles, can determine the extent of brain and nerve damage exhibited by patients.

Despite the extensive research dedicated to deciphering AD pathogenesis and discovering novel drug treatments, the comorbid nature of this disease, along with other psychological and physiological complications, obstructs the ability to examine the therapeutic effectiveness of these methods. AD pathogenesis is initially isolated to the limbic region in afflicted patients, but as the disease progresses to other neocortical areas, additional cognitive symptoms manifest and become apparent^3^. Due to the long prodromal period of AD, the potential for early diagnosis of AD is crucial to effectively utilize disease-modifying drugs. However, the dearth of such treatments can be attributed to the fact that most therapeutic attempts are rendered ineffective due to the advanced progression of the disease. Thus, in order for a drug to be successful in combating AD, the extent of disease progression at the time of treatment must be considered in tandem with the pathophysiological target and composition of the drug^4^.

**Fig. 1:**
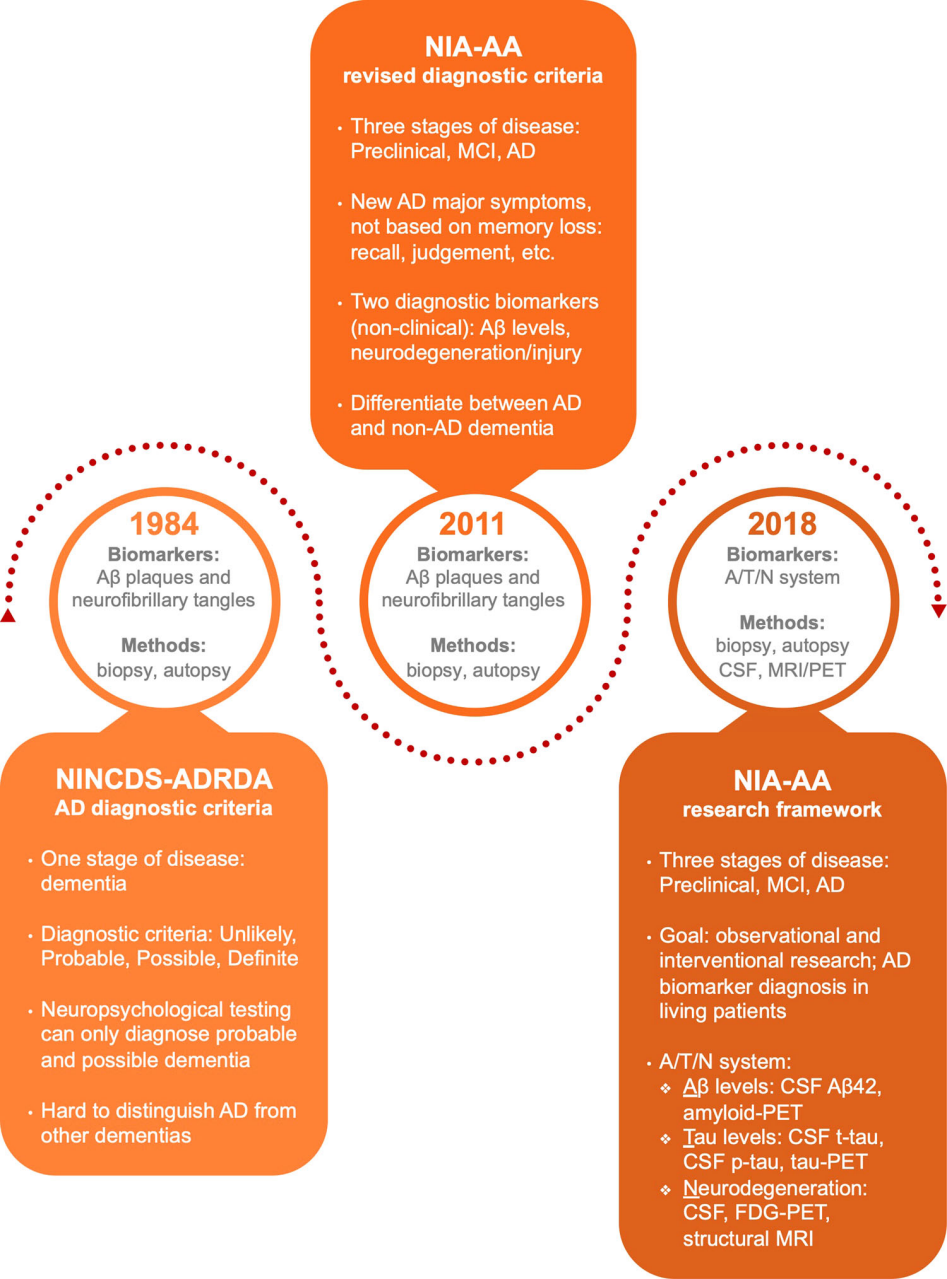
Evolution of AD diagnostic criteria. A timeline of revisions applied to the protocols for AD diagnosis. As the understanding of AD pathology has developed, the criteria for diagnosis have reflected this expansion of knowledge. The clinical diagnostic standard underwent two major revisions after its initial publication in 1984, with the latest revision occurring in 2018

The criteria for diagnosing AD published by the National Institute on Neurological and Communicative Disorder and Stroke and the Alzheimer’s Disease and Related Disorders Association (NINCDS-ADRDA) have been widely utilized by clinicians to distinguish between the symptoms present in “probable,” “possible,” or “definite” AD^5^. After the diagnostic criteria for AD were released in 1984, they were updated in 2011 by the National Institute on Aging and Alzheimer’s Association (NIA-AA) due to the advancement of knowledge regarding AD pathogenesis and considerable advancements in modern clinical, imaging, and research technologies (Fig. 1). One significant change was the inclusion of imaging and cerebrospinal fluid (CSF) biomarkers as secondary diagnostic tools to confirm the origin of clinical dementia symptoms exhibited by probable AD patients. These tools confirmed that the symptoms were solely correlated with AD pathophysiological pathways and did not originate from comorbid diseases. However, under this revision, biomarkers could only be utilized as supportive diagnostic tools in clinical research and could not be implemented in clinical diagnostic settings due to the insufficient standardization of the analytical results, the limited availability of the tools, and a lack of evidence correlating biomarker concentrations with AD pathology. Although the new guidelines aided in accurately diagnosing AD patients, the standardized criteria relied on the expression of symptoms, which were only apparent once AD pathology had reached advanced stages. Thus, early diagnosis or disease prediction was not possible because this protocol only confirmed the presence of AD^6^.

Due to an increase in evidence supporting the existence of a long prodromal period in AD pathogenesis, there has been a paradigm shift in the objective of diagnostic techniques from confirming the presence of symptomatic AD to identifying the disease in its asymptomatic stages. This shift is apparent in the 2018 revision of the NIA-AA diagnostic criteria, which identified imaging and CSF biomarkers as valid diagnostic tools (Fig. 1). Hence, establishing biomarkers as reliable reflections of disease progression has permitted their use as diagnostic tools for definitively diagnosing AD and reduced the dependence on patient biopsies or autopsies to obtain a definitive diagnosis. Within the new protocol, the A/T/N classification system for AD biomarkers, in which “A” represents Aβ biomarker concentrations, “T” refers to the level of tau biomarkers, and “N” reflects neurodegeneration biomarkers or neuronal injury, was introduced. This new arrangement distinguishes the three biomarker groups by the pathological mechanism that each partakes in (aggregated Aβ peptides, aggregated tau proteins, or neurodegeneration/neuronal injury)^7^.

The antemortem diagnosis of AD can be typically classified into two categories: brain imaging and liquid biopsy. Imaging biomarkers utilize magnetic resonance imaging (MRI) and positron emission tomography (PET) to analyze brains afflicted with AD, while fluid biomarkers can be obtained from biological fluids, such as CSF, blood, tears, saliva, and others. Brain imaging plays a critical role in diagnosis because neurodegeneration often parallels and precedes the cognitive decline that is symptomatic of AD. The four types of imaging modalities are structural MRI, functional MRI, ^18^F-2-fluoro-2-deoxy-D-glucose (FDG) PET, and amyloid-PET. Structural or compositional abnormalities can be monitored with MRI scans, while FDG-PET monitors glucose metabolism mechanisms to identify areas of decreased brain activity. Of the various imaging methods, amyloid-PET is the most reliable diagnostic imaging tool because of its ability to characterize aggregated Aβ within the brain by utilizing amyloid tracers. Although imaging biomarkers are approved for clinical use and are considered advantageous due to their reliability in accurate diagnoses, the economic burden and accessibility issues associated with these imaging modalities continue to impede their comprehensive use in identifying AD. In addition to these difficulties, MRI and FDG-PET scans often struggle to distinguish AD from other neurodegenerative disorders^8^.

On the other hand, fluid biomarkers exhibit unique diagnostic advantages that are not available with imaging biomarkers. In comparison to MRI and PET scans, fluid biomarkers are more accessible and affordable. As possible candidates for the preventative screening of at-risk individuals and additional attempts to diagnose AD in its early stages, imaging modalities may impose significant burden on patients due to the exorbitant costs and health hazards associated with utilizing the techniques for diagnostic examinations. Moreover, fluid biomarker concentrations may not result in noticeable fluctuations in early stages of disease pathogenesis, so a combination of methods is warranted to enhance diagnostic accuracy^9^. Therefore, when observing changes in various fluid biomarker concentrations, it is advantageous to incorporate clinically approved imaging modalities to confirm the accuracy of the diagnostic results. Although evaluating imaging biomarkers in tandem with fluid biomarkers may provide enhanced diagnostic results, this review will focus on the ability of fluid biomarkers to accurately reflect AD pathology and their potential as clinical diagnostic tools. Imaging modalities, such as PET, have been beneficial in aiding diagnosis, and with a shift in focus from disease confirmation to asymptomatic detection, fluid biomarkers have become increasingly attractive for clinical use. Thus, it is imperative to review the recent advancements pertaining to fluid biomarkers as AD diagnostic tools to aid their integration in clinical settings.

## Main text

### Cerebrospinal fluid

Since CSF resides in the subarachnoid space and ventricular system of the brain and spinal cord, biochemical changes that occur in the brain are reflected in the CSF. This mirroring of the environments allows for the implementation of CSF as a diagnostic tool for various pathologies, such as infectious diseases and autoimmune disorders. Hence, CSF is an ideal candidate to identify potential AD biomarkers without the need for autopsy or biopsy due to its ability to depict AD pathology in parallel to its progression. The biomarkers that are the most indicative of AD are Aβ, total tau (*t*-tau), and phosphorylated tau (*p*-tau). These three biomarkers have risen in prominence as the analysis of patients' CSF has become feasible. In most research regarding CSF biomarkers, the concentrations of Aβ and tau isoforms are analyzed independently or in comparison with one another.

The aggregation of Aβ into amyloid plaques and tau into neurofibrillary tangles within brain tissues is associated with AD pathology. Although various isoforms of Aβ can be identified in AD patients, the CSF levels of Aβ40 and Aβ42 are the most reliable indicators of the disease. This amyloidogenic protein is detected throughout the human body, but the concentrations of Aβ42 in patients' CSF often correlate with Aβ levels in the brain^1^. With the introduction of amyloid-PET for longitudinal brain imaging, multiple clinical studies have established reduced levels of CSF Aβ42 as an indicator of its presence in fibrils and plaques in AD brains^10–14^. This decrease in CSF Aβ42 concentration can be attributed to the fact that, as Aβ42 aggregates into fibrils and plaques in the brain, a lower amount of the peptide is able to diffuse into the CSF.

Another derivative of Aβ that can act as a potential AD biomarker is Aβ40, which is the most abundant isoform present in the CSF. Although there are no substantial changes in the levels of CSF Aβ40 in AD patients and the CSF concentration of Aβ40 does not correlate with amyloid deposits in the brain, a significant decrease can be seen when amyloidogenic peptide levels are compared by the CSF Aβ42/Aβ40 ratio^15–22^. This ratiometric analysis is more reliable than solely observing Aβ42 concentrations because it compensates for intraindividual fluctuations within AD patients. Truncated forms of this amyloidogenic peptide, such as Aβ37, Aβ38, and Aβ39, can be comparatively analyzed to provide a more accurate reflection of AD pathology. Of these fragmented peptides, Aβ38 tends to exhibit increased concentrations in patients' CSF. Thus, the ratio of Aβ42/Aβ38 has been shown to positively correlate with imaging biomarkers and provide a stronger association with AD pathogenesis than CSF Aβ42 alone^15,22–26^. A recent study involving three different cohorts, established the efficacy of these ratiometric analyses in accurately diagnosing and distinguishing AD from other forms of dementia^15^. Due to the ability of these amyloidogenic peptide ratios to correlate with disease progression, the concentrations of Aβ42 and the ratio comparisons of Aβ42/Aβ40 are accepted as candidate AD biomarkers in the 2018 revision of the NIA-AA diagnostic guideline^7^.

Increases in tau protein concentrations in patients' CSF have also been correlated with AD^27,28^. Tau proteins are referred to as either *p*-tau, which indicates hyperphosphorylated tau proteins, or *t*-tau, which consists of various tau protein isomers. Multiple studies have established a correlation between CSF *p*-tau levels and the formation of neurofibrillary tangles in the brain^13,29–31^. On the other hand, CSF *t*-tau concentrations indicate the severity of neurodegeneration and neuronal or axonal damage in AD patients' brains^32,33^. However, fluctuations in CSF *t*-tau levels also occur in acute disorders, such as stroke and brain trauma, and chronic neurodegenerative disorders, such as Creutzfeldt-Jakob disease. Therefore, it is less specific for indicating AD pathology and more indicative of overall brain degeneration and dysregulation. Although tau proteins are not AD-specific biomarkers, integrating disease-specific and nonspecific biomarkers have been shown to be an effective diagnostic tool. For instance, a study comparing the concentrations of tau proteins and Aβ reported the capability of the CSF tau (*t*-tau or *p*-tau)/Aβ ratio in detecting AD pathology in its early stages^34^.

Recent findings have established the use of Aβ^35^ and tau^36–40^ as valid proxies for neuropathological changes related to AD progression. However, these studies confirmed diagnoses in advanced stages of the disease. Aβ oligomers, which are found in earlier stages of pathogenesis and inhibit long-term potentiation, may play a crucial role as potential early diagnostic targets. Although there is extensive research that has attempted to establish the reliability of these candidate biomarkers, no standard has been codified due to the contradictory results regarding the concentration of these proteins needed to accurately diagnose AD in the prodromal stages. For instance, CSF Aβ42 concentrations have been found to increase^41,42^, decrease^43–48^, or experience no significant change^49–54^ as cognitive functions deteriorate.

In addition to this discrepancy, CSF biomarkers are problematic due to the invasive nature of sample procurement. Lumbar puncture results in discomfort or pain because of the larger and longer needle required compared to that used in regular intravenous punctures, and anesthesia cannot be provided to the patient as to avoid the possibility of contaminating the CSF. It is also difficult to repeatedly and routinely check patients' CSF due to the limited availability of CSF and possible health complications that can arise when sampling is conducted too frequently. However, compared to imaging biomarkers, those acquired from the CSF are advantageous because they present a low economic burden, are more accessible, and do not involve exposure to radioactivity. Therefore, integrating the diagnostic capabilities provided by both imaging and CSF biomarkers will allow for enhanced analyses within clinical settings.

### Blood

In order to identify at-risk AD patients, it must be possible to routinely screen for the disease before symptoms appear. Because AD is asymptomatic during its prodromal period, it is difficult for patients to partake in preventative screening measures that are economically and physically burdensome. The accessible and inexpensive nature of blood collection makes it an attractive candidate for early disease detection and diagnosis. It does not require the type of specialized diagnostic machines that are required to detect imaging biomarkers nor is it as intrusive as CSF collection. Blood biomarkers are already implemented in diagnostic procedures for cardiovascular and cancer patients, so they could play an imperative role as preventative screening measures in the early detection of AD^55^. Biomarkers obtained from blood samples can act as surrogate markers, which are capable of indirectly indicating the pathological progression of a disease. However, the filtering mechanism of the blood-brain barrier (BBB) prevents the diffusion of substances into the blood; thus, the detection of blood biomarkers exhibits lower sensitivity and specificity than that of biomarkers obtained from patients' CSF.

Blood biomarker research seemed futile due to contradictions among research findings and difficulties in replicating results. To address these issues and because of the impression that variations in preanalytical research conditions may have contributed to such anomalies, a standardized guideline for blood biomarker research was established^56^. The guideline consolidated detailed information to reduce minor discrepancies and obtain more accurate results. They proposed methods to standardize the effects of controllable variables such as blood sample collection locations and times, sample treatment procedures, needle sizes, collection vessel types, centrifugation parameters, the duration of time between sampling and freezing, the number of freeze-thaw cycles, and aliquot sizes. Although differences in data analysis may cause slight variations in results, the standardization of preanalytical techniques allows for the possibility of result validation and cross-validation. The implementation of this standardized procedure in research studies has allowed blood biomarkers to be applied as a method for the clinical diagnosis of patients.

For a biomarker to be validated for clinical use, it must be able to reliably reflect its role concomitant with disease pathogenesis. Aβ is largely supported as the earliest existing AD species and is an attractive candidate as a blood biomarker because it can easily penetrate the BBB. However, it has yet to be accepted as a reliable indicator in blood analyses due to inconsistent research results. Numerous studies dedicated to investigating the levels of Aβ in individuals susceptible to AD have reported that Aβ40^57–59^ or Aβ42^58^ levels are elevated or Aβ40^60^ or Aβ42^59,61^ concentrations are reduced in patients susceptible to AD or that Aβ40^61,62^ and Aβ42^57,62^ levels are irrelevant in determining a patient’s risk of dementia. These varying results could be due to analytical interferences, such as epitope masking caused by hydrophobic Aβ binding to plasma proteins. However, such adverse findings could also be attributed to the fact that there are many uncertain factors concerning the source of plasma Aβ. Some suggest that they are produced by the proteolytic cleavage of APP located in peripheral tissues^1^. APP is expressed in various cells throughout the body; thus, the cellular origins of Aβ deposits in the brain and cerebral vessels are unknown. Studies have postulated that cerebral amyloid deposits may be derived from the periphery, while others have suggested that amyloid deposits in cerebral vessels may originate from circulating Aβ peptides^63–67^.

Despite the ambiguous nature of the origins of Aβ, it is well established that low-density lipoprotein receptor-related protein-1 is able to transport Aβ from the brain across the BBB to the blood. Thus, the emergence of standardized research guidelines and the ability to utilize PET amyloid imaging using Pittsburgh Compound B (PIB-PET) in the selection of patients for experimental studies has aided in establishing a correlation between plasma Aβ species and brain amyloid deposition. Plasma Aβ and Aβ-approximate peptide concentrations were reported to be consistent with amyloid-PET results with a sensitivity and specificity of 0.93 and 0.96, respectively^68^. Another study utilized the plasma APP/Aβ42 and Aβ40/Aβ42 ratios to reliably predict brain Aβ burden, obtained through PIB-PET, to approximately 90% accuracy^69^.

Plasma is composed of countless proteins other than amyloidogenic peptides. Multiple studies have attempted to determine new proteins capable of classifying and predicting AD. A study in 2007 analyzed ~120 plasma proteins and discovered 18 signaling proteins capable of differentiating between AD, mild cognitive impairment (MCI), and normal cognition in control subjects^70^. The researchers were able to accurately diagnose 90% of AD patients and 91% of MCI patients. This study was the first to reveal the potential for blood biomarkers as diagnostic tools, but such results have been difficult to replicate. However, at the Texas Alzheimer Research and Care Consortium in 2011, an analysis of 30 serum proteins showed 88% sensitivity and 82% specificity for AD^71^.

In addition to the issue of reproducibility, there is skepticism regarding the efficacy of these potential biomarkers as early diagnostic tools because protein panel research analyzes plasma components obtained from patients who already exhibit clinical symptoms. In addition, AD is comorbid with various other neurodegenerative diseases and vascular risk factors; thus, the presence of these variables may affect the research results obtained. Although it is reasonably sound to study blood plasma proteins as possible diagnostic markers, numerous internal and external patient factors, such as personality and environment, must be considered to eliminate any possible variations resulting from peripheral interferences that are reflected in the blood.

### Oral fluids

Other than CSF and blood, there is an array of biofluids, such as oral, ocular, and olfactory fluids, used in clinical settings to detect and monitor various disorders. Although blood sampling is less invasive than CSF sampling, periodic monitoring conducted with these easily accessible biological fluids would make comprehensive diagnostic procedures more convenient and noninvasive. However, there are low concentrations of proteins in these fluids, so enhanced sensitivity is required for detection. Similar to blood biomarkers, these alternative fluids can be categorized as surrogate markers.

Due to the straightforward and noninvasive nature of salivary gland secretions, biomarkers obtained from this biofluid are beginning to gain traction as an emerging target for detecting diseases in early stages. The growing prominence of salivary diagnostics has unveiled biomarkers for various diseases and cancers. The ease of collection and analysis permits clinicians to implement point-of-care diagnostics outside of the laboratory, thus allowing for more accessible and harmless methods of preventative screening. Recent studies analyzed saliva samples from 15 AD patients and found that the salivary levels of Aβ42 were significantly higher than those in controls^72–74^. However, further experiments with larger sample sizes and those that involve the implementation of this experimental method for the potential detection of other neurological conditions, such as MCI and Parkinson’s disease, are pending. Another study successfully profiled saliva metabolites obtained from normal, MCI, and AD subjects with ^1^H NMR metabolomics^75^. Although this field of research has only recently emerged, the development of reliable and sensitive salivary biomarkers would be significant as an early periodic screening tool for those susceptible to AD.

### Ocular fluids

Aβ aggregates have also been discovered in the ocular region of AD patients, namely, the lens^76–78^ and retina^22,79–82^. The human eye, which permits the noninvasive observation of disease pathology mechanisms that may be similarly reflected in the afflicted brain, is an extension of the central nervous system and shares structural similarities with the brain. In a 2010 study, researchers were able to establish that the ocular lens provides an ideal environment for Aβ aggregation due to the absence of the vascular systems and nerves^78^. However, the lack of these structures raises the question of whether aggregated Aβ found in these regions is associated with AD pathogenesis. On the other hand, the retina is composed of central nervous system neurons and is vascularized, permitting it to be a more attractive candidate to allow the observation of disease mechanisms and record the presence of ocular biomarkers. In a 2017 study, researchers were able to observe and map Aβ deposits that reflected the pathology exhibited within AD brains in the retina^83^. This study was able to detect retinal Aβ plaques 2 months prior to their presence in the hippocampus and cortex of AD murine models. In addition to being noninvasive and inexpensive, the detection of ocular biomarkers allows for the possibility of preliminary AD diagnosis in the comfort of the individual’s own home through the use of mobile ocular imaging modalities^84^. Targets identified in the ocular fluid may play a crucial role as noninvasive and economical surrogate AD biomarkers, especially because it is the only extension of the central nervous system that is not structurally restricted by the skull, allowing it to be easily observed.

### Olfactory fluids

As the focus shifts from mitigation to prevention, olfactory disorders have been increasingly identified in a variety of neurological disorders. In AD, neurofibrillary tangles have been isolated from various locations throughout the olfactory system. Studies have discovered neurofibrillary tangles in the noses of AD patients; similarly, researchers have observed increased concentrations of *t*-tau and *p*-tau in nasal secretions^85–87^. A study conducted in 1987 discovered neuritic plaques, neurofibrillary tangles, and neuropil threads in the olfactory bulbs of AD patients^86^. Tau tangles were observed in the anterior olfactory nucleus and throughout the olfactory bulb, excluding the outer layer. On the other hand, Aβ plaques were solely situated in the anterior olfactory nucleus. The most recent study established that tau protein concentrations can be detected in the nasal secretions of AD patients^87^. Although this study was preliminary in nature, the capacity to identify tau isoforms within this abundant and readily accessible fluid permits the possibility of monitoring and diagnosing at-risk patients more efficiently and effectively.

## Conclusion

Although various fluid biomarkers were discussed in this review, none has yet been established or implemented in early diagnostic protocols. AD is still considered to be an untreatable and incurable disease because, by the time symptoms appear in patients, the disease has progressed to a point where most therapeutic agents are rendered ineffective. Most drug trials target biomarkers indicative of AD; however, it is difficult to discover effective therapeutic drugs since the field has yet to establish a consensus on the concentrations of the respective biomarkers required for diagnosis. Accurate experimental results are necessary to identify suitable biomarkers, which will provide accurate disease diagnosis by differentiating between AD and its comorbid ailments. Although imaging biomarkers are already widely utilized in clinical diagnosis, their use is an economic burden to patients, requires special facilities that can accommodate expensive machines, and exposes patients to radioactive particles. In addition to these detriments, imaging biomarkers can only be utilized to confirm AD diagnosis once symptoms are present.

Successful early diagnosis entails the ability to detect AD in asymptomatic patients. The ability to screen for AD in its early stages would provide preventative therapeutic methods and reduce the economic burden that accompanies diagnosis, such as treatment and patient care. Ideal surrogate biomarkers would be accessible, affordable, and abundant so that they could be implemented into point-of-care testing and thus allow the reliable diagnosis of at-risk patients. Studies dedicated to establishing biomarkers obtained from CSF, blood, and other bodily fluids have demonstrated the individual potential of these biomarkers (Table 1) and have delineated the correlations between AD and the biomarkers obtained from differing fluids^88,89^. Most research pertaining to these varying biomarkers utilizes similar methodologies despite the difference in fluid mediums. For instance, immunoassays are commonly used in quantifying biomarker concentrations in CSF, plasma, oral, and ocular samples. In addition to this quantification method, mass spectrometry is implemented to detect surrogate biomarkers in aqueous solutions when there are diverse impurities. Other techniques often employed in analyzing fluid biomarkers are nuclear magnetic resonance, microscopy, and optical coherence tomography, which is solely used to detect markers in the eye. Although there are many methodologies available, their compatibility and accessibility for use within medical institutes should be considered. Until recently, immunoassays have been the most prevalent techniques used to fulfill these criteria.

**Table 1:** Fluid biomarker sampling sites for Alzheimer’s disease diagnosis and research

Fluid	Biomarkers	Methodology	Results	Refs.
CSF	Aβ42	Commercial ELISA	CSF Aβ42 has an inverse correlation with amyloid burden, as measured by PIB-PET	^10– 14^
		Commercial immunoassays	Ratios of CSF Aβ42 to other Aβ isoforms (Aβ40 or Aβ38) are strongly correlated with accurate AD diagnosis	^15– 26^
	*t*-Tau	Commercial ELISA	*t*-Tau is associated with neurodegeneration and neuronal/axonal damage	^32, 33^
	*p*-Tau	Commercial ELISA	*p*-Tau has a positive correlation with NFT regional distribution	^13, 29– 31^
Whole blood	120 proteins	Commercial cytokine antibody array assay	18 out of 120 proteins can be used to diagnose AD patients with 90% accuracy and predict MCI progression to AD with 91% accuracy	^70^
	30 proteins	Multiplexed immunoassay humanMAP	30 candidate markers can accurately diagnose AD with 88% sensitivity and 82% specificity	^71^
Plasma	Aβ40	Commercial sandwich ELISA	Plasma Aβ40 in AD patients increases (57–59), decreases (60), or is irrelevant (61,62)	^57– 62^
		IP-MS with MALDI-TOF mass spectrometry	Ratios of plasma Aβ40/Aβ42 and APP/Aβ42 correlate with amyloid burden, as measured by PIB-PET	^69^
	Aβ42	Commercial sandwich ELISA	Plasma Aβ42 in AD patients increases (58), decreases (59, 61), or is irrelevant (57,62)	^57– 59, 61, 62^
		Two-step immunoassay	Plasma Aβ42/Aβ40 ratios correlate with AD diagnosis	^90, 91^
Oral	Aβ42	Commercial ELISA	Salivary Aβ42 is significantly elevated in AD patients	^72– 74^
	Metabolites	NMR spectroscopy	There are several candidate AD biomarkers in saliva	^75, 92^
Ocular	Aβ	FLES	Ocular Aβ levels correlate with quantitative PET and predict AD	^93^
Ocular—retina	Aβ plaques	Curcumin staining	Retinal Aβ plaques are present in AD patients and mice	^79, 81, 83^
	RNFL	Optical coherence tomography	AD patients tend to have a reduction in RNFL thickness	^80^
	Aβ42	Immunohistochemistry	Compared to the brains of AD mice, the retinas exhibit lower Aβ production	^82^
Ocular—lens	Aβ40, Aβ42	ESI-MS	Aβ42 and Aβ40 is found in the lenses of postmortem AD patients	^77^
	Aβ aggregates	Commercial ELISA	Aβ potentiates lens protein aggregation and can accumulate in the lens similar to how it accumulates in the cerebrum	^78^
Ocular—aqueous humor	Aβ40	SELDI-MS protein array chip	Aβ40 is present in the primary aqueous humor of AD patients	^77^
Olfactory	Aβ	Histopathology	Few neuritic plaques are present in the anterior olfactory nucleus; thus these plaques do not correlate with NFTs	^85, 86^
	Tau	Histopathology	NFT/neuropil threads are present in the anterior olfactory nucleus and olfactory bulb (except the outer layer)	^86^
		Commercial olfactory test	*t*-Tau and *p*-tau are present in AD patient nasal secretions	^87^

*Aβ* amyloid-β, *t-tau* total tau, *p-tau* phosphorylated tau, *NFTs* neurofibrillary tangles, *ELISA* enzyme-linked immunosorbent assays, *PET* positron emission tomography, *humanMAP* human multi-analyte profile, *IP-MS* immunoprecipitation-mass spectrometry, *MALDI-TOF* matrix-assisted laser desorption ionization–time-of-flight, *NMR* nuclear magnetic resonance, *SELDI-MS* surface-enhanced laser desorption ionization mass spectrometry, *ESI-MS* electrospray ionization mass spectrometry, *FLES* fluorescent ligand eye scanning, *RNFL* retinal nerve fiber layer

CSF biomarkers exhibit the highest sensitivity and specificity because they directly interact with the brain. Although Aβ40, Aβ42, Aβ42/Aβ40, *t*-tau, and *p*-tau were categorized as CSF biomarkers in the revised NINCDS-ADRDA, their detection induces physical and financial burden. In addition to these adversities, CSF sampling requires special techniques and tools, so it is not widely accessible. On the other hand, blood biomarkers are attractive as early markers of AD pathogenesis due to the accessible and inexpensive nature of blood. Despite the fact that blood biomarkers are established diagnostic methods for diabetes and cancer, markers specific to AD exhibit decreased specificity and sensitivity in comparison to the analytical results obtained for CSF biomarkers. Regardless, research endeavors are ongoing due to the positive potential they possess as alternative diagnostic candidates. Another disadvantage of blood biomarkers lies in the controversial findings regarding the correlations between their concentrations and AD progression. However, recent endeavors have successfully associated AD pathogenesis with Aβ concentrations and plasma protein panels.

Due to the noninvasive and low-cost sampling measures of oral, ocular, and olfactory fluids, biomarkers obtained from these systems have appeared as potential diagnostic tools. While blood biomarkers struggle to provide an accurate analysis, these alternative fluid biomarkers may be used in tandem with other clinical procedures to enhance diagnosis. These surrogate markers are promising and are currently in the preliminary stages of research; therefore, their efficacy and accuracy are difficult to determine. However, implementing a combination of these candidate fluid biomarkers into diagnostic settings may potentially permit the identification of at-risk patients during asymptomatic stages.

AD is the most prevalent form of dementia and is the leading cause of mortality in the elderly. Disease-modifying therapies are most effective in the asymptomatic stages of disease progression; thus, it is imperative to develop an early diagnostic procedure that integrates imaging biomarkers with fluid biomarkers to provide a convenient and accessible screening system for seniors or individuals with familial AD. Currently, CSF biomarkers are the only variety of fluid biomarkers utilized in the early diagnosis of AD, but they cannot be implemented as a preventative screening system due to their limited accessibility and the invasive nature of CSF collection. Although the correlations of AD with alternative biomarkers obtained from other biological fluids is uncertain, the convenience and practicality of these biomarkers would allow for the preventative screening of individuals susceptible to this neurodegenerative disease. Thus, it is essential to establish and develop candidate fluid biomarkers to shift the treatment of this neurodegenerative disorder from alleviation to prevention.
